# Methods for improved bileaflet aortic valve detection prior to transcatheter aortic valve replacement

**DOI:** 10.3389/fcvm.2026.1755835

**Published:** 2026-02-23

**Authors:** Justin T. Tretter, Mackram F. Eleid, Francesco Bedogni, Josep Rodés-Cabau, Ander Regueiro, Luca Testa, Shmuel Chen, Attilio Galhardo, Kenneth A. Ellenbogen, Martin B. Leon, Shlomo Ben-Haim

**Affiliations:** 1 Hobart Healthcare Research Institute, London, United Kingdom; 2 Department of Cardiovascular Medicine, Mayo Clinic, Rochester, MN, United States; 3 Department of Cardiology, Policlinico San Donato, Milan, Italy; 4 Department of Cardiology, Quebec Heart and Lung Institute, Laval University, Quebec City, QC, Canada; 5 Cardiology Department, Hospital Clínic de Barcelona, Barcelona, Spain; 6 Division of Cardiology, NewYork-Presbyterian/Weill Cornell Medical Center, New York, NY, United States; 7 Division of Cardiology, Virginia Commonwealth University School of Medicine, Richmond, VA, United States; 8 Division of Cardiovascular Medicine, Columbia University Irving Medical Center/New York-Presbyterian Hospital, New York, NY, United States; 9 Cardiovascular Research Foundation, New York, NY, United States

**Keywords:** bicuspid aortic valve, calcific aortic valve disease, complete heart block, computed tomography, permanent pacemaker implantation, transcatheter aortic valve replacement

## Abstract

**Background:**

Bileaflet aortic valve prevalence in transcatheter aortic valve replacement (TAVR) patients is poorly defined. We evaluated a TAVR cohort to determine the bileaflet aortic valve prevalence and understand features which may improve detection. In addition, we related valvar morphology to the occurrence of permanent pacemaker implantation (PPI) following TAVR.

**Methods:**

Aortic valvar morphology diagnosis was recorded from the pre-procedural cardiac CTA reports prior to TAVR. Commissural angles, comparison of commissural heights, and dynamic visual inspection of the aortic valve were subsequently evaluated on pre-procedural cardiac CTA by an expert cardiac anatomist and imager, methods previously validated in a surgical cohort, to determine aortic valvar morphology and compared to the historical diagnosis. Relationships between valvar morphological characteristics with the need for PPM within 30-days post-TAVR were determined.

**Results:**

Four-hundred and thirty-three (mean age 81.3 ± 6.6 years, 53.8% female) underwent TAVR [corrected diagnosis: 393 (90.8%) trileaflet vs. 40 (9.2%) bileaflet valves]. Bileaflet valves were historically misdiagnosed in 80% of pre-procedural cardiac CTA reports. Thirty-four (85.0%) had intercoronary leaflet fusion [mean commissural angle = 148.1 (18.3) degrees]. A commissural angle threshold of 141.1 degrees had a sensitivity of 0.73 and specificity of 0.86 for identifying a bileaflet valve. PPI post-TAVR occurred in 38% bileaflet vs. 19% trileaflet patients (*p* *=* *0.0114*) [unadjusted OR for bileaflet valve requiring PPI = 2.54, 95% CI (1.25–5.01)].

**Conclusions:**

Bileaflet aortic valves are commonly misdiagnosed. Assessment of the commissural angle and comparison of commissural heights may improve CTA-based diagnostic accuracy prior to TAVR. Improved detection may guide improved outcomes in this higher risk population.

## Graphical Abstract

**Figure:**
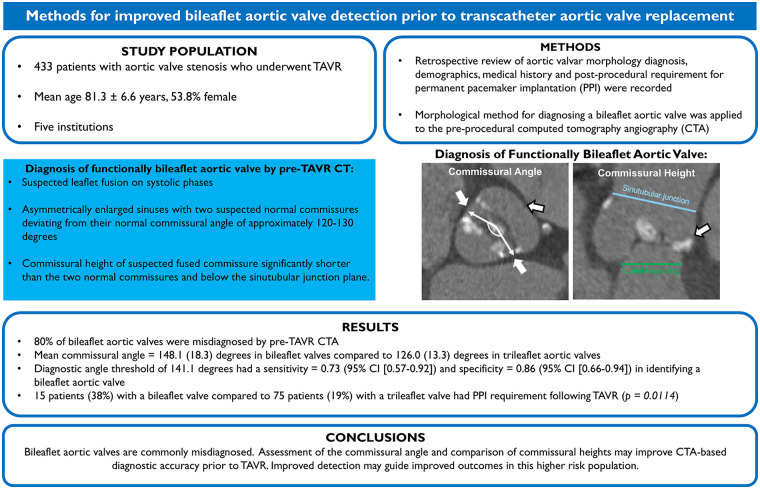
Improved bileaflet aortic valve detection can be achieved by cardiac computed tomography prior to transcatheter aortic valve replacement.

## Introduction

A bileaflet (bicuspid) aortic valve accounts for up to 10% of elderly patients currently treated by transcatheter aortic valve replacement (TAVR) (1–4). Related to TAVR, these congenitally malformed valves, commonly compounded with calcific disease, are more technically demanding with increased risks (5). However, various retrospective and observational studies have reported discrepant findings (6). For example, the rate of need for permanent pacemaker implantation (PPI) following TAVR with newer-generation prostheses in those with a bileaflet aortic valve has been reported between 15%, to over 40% (5, 6). This may partly be influenced by institutional volume and experience (5, 7). These discrepancies may also relate to the high misdiagnosis rate of bileaflet valves prior to the TAVR procedure. In fact, a single center study reported misdiagnoses of bileaflet valves in up to 90% of elderly patients considered for TAVR when relying on transthoracic echocardiogram (8). While the use of cardiac computed tomography angiography (CTA), magnetic resonance angiography, or transesophageal echocardiogram may approximately double the detection rate, a large proportion of these patients remain misclassified going into the TAVR procedure (8, 9).

Advancements in the imaging interrogation and surgical strategies towards repairing the bileaflet aortic valve have necessitated improved descriptions and classification schemes (10–12). Historical classification schemes solely focused on the number of leaflets and presence or absence of leaflet fusion with corresponding raphe (13). Newer classification schemes hold in common the additional description of the commissural angle between the two normal commissures in the more prevalent functionally bileaflet aortic valve which retains three sinuses (12, 14). In this form of the bileaflet valve, the commissural angle has a predictable relationship to both the degree of leaflet fusion and the height of the corresponding fused commissure and its underlying hypoplastic interleaflet triangle (12, 15). Furthermore, morphologic and surgical observational studies suggest that over 80% of those with a functionally bileaflet aortic valve may have abnormal commissural angles with resulting leaflet and sinus asymmetries when compared to a normal trileaflet valve (16). This feature alone may aid the clinician in detecting a bileaflet aortic valve (12). The remaining 10%, however, may appear indistinguishable from a trileaflet valve with commissural angles approximating 120 degrees (15). Interrogation of the commissural height of the suspected fused leaflet will aid in properly diagnosing these rarer subtypes of functionally bileaflet aortic valves (10–12, 17). This understanding may be used to improve the detection rate of bileaflet aortic valves (10, 17). In fact, in a recent report utilizing CTA for personalized surgical planning and execution in children and adults with congenitally malformed aortic valves, this approach was validated by intraoperative inspection, reporting approximately two-thirds of patients with a functionally unileaflet aortic valve had been misclassified prior to this pre-surgical imaging evaluation (18). Understanding both commissural angles and commissure height has also proven to be an important variable to dictate the surgical repair strategy and predict subsequent repair durability (15, 19).

While these described nuances have become common place in interrogation and surgical planning for those with a bileaflet aortic valve (11, 18), little attention has been given to those considered for TAVR. In the current study we aimed to retrospectively assess these previously validated features by cardiac CTA in a large population considered for TAVR to the proportion of patients with a bileaflet aortic valve. Furthermore, we aimed to assess these morphological valvar features which may aid in the detection rate of these congenitally malformed valves and related valvar morphology to the occurrence of need for PPI following TAVR.

## Methods

### Study population

This study included patients who underwent TAVR from five institutions between November 2021 and May 2024 and were evaluated prior to the procedure by gated cardiac CTA. Patient selection for TAVR was performed per local standard practice. Study exclusion criteria included: low-quality images related to cardiac motion or respiratory artifact, scans which did not utilize cardiac gating, and valve-in-valve procedures. Patient demographics, medical history and comorbidities were recorded. This study was approved by the institutional review boards of the participating centers with informed consent required for patient involvement.

### Pre-procedural cardiac computed tomography acquisition and assessment

Routine preprocedural cardiac CTA for TAVR was obtained per clinical guidelines at the discretion of the acquiring institution (20). Scan acquisition included a cardiac retrospective ECG-gated contrast-enhanced data set acquired throughout the cardiac cycle or ECG-gated dataset acquired in peak systole of the aortic root and heart.

**Figure 1:**
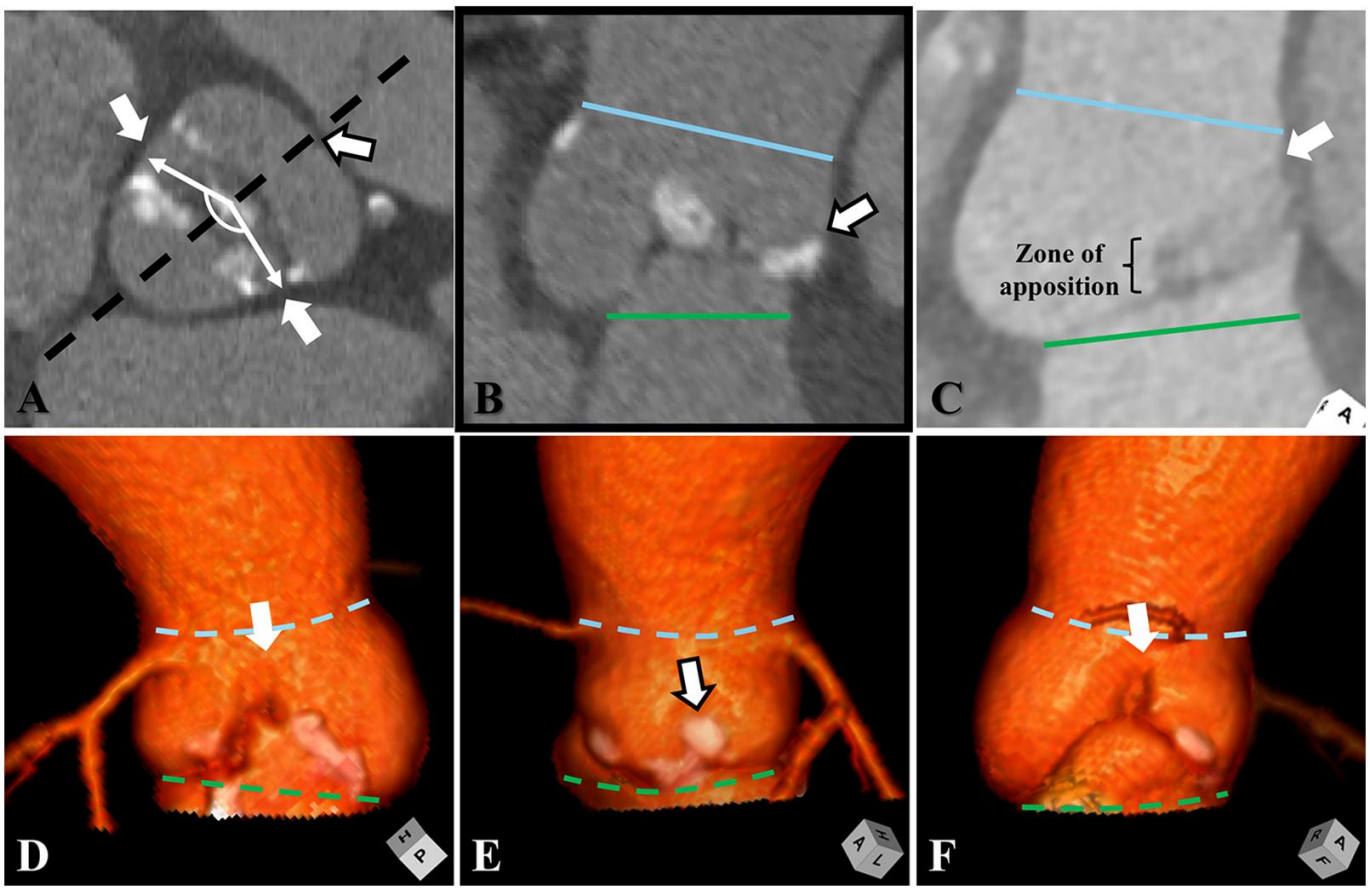
Cardiac computed tomographic assessment of the commissural angle and commissural heights are demonstrated. **(A)** Short axis of the aortic root is obtained to measure the commissural angle (angulated white double-headed arrow). In this functionally bileaflet aortic valve with fusion between the coronary leaflets it measures approximately 170 degrees. The black hashed line represents the center bisecting plane, which cuts from the interrogated commissure across to the midline of the opposite leaflet. **(B)** In the center bisecting plane, the height of the fused commissure (white arrow with black outline) above the plane of the virtual basal ring (green lines and hashed lines) will be short relative to the sinutubular junction (blue lines and hashed lines) and relative to interrogation of the normal commissures. **(C)** The long axis of the center bisecting plane is demonstrated in a normal trileaflet valve depicting a double shadow of the interrogated zone of apposition, leading up to the normal commissure (white arrow). **(D–F)** Three-dimensional reconstructions of the depicted bileaflet valve demonstrate three sinuses, with a shortened interleaflet triangle (white arrow with black outline) and commissural height of the fused coronary leaflets relative to the other two normal commissures (white arrow).

The cardiac CTA was retrospectively assessed by an expert cardiac anatomist and imager (J.T.T), the same individual who had previously validated this approach compared to direct intraoperative surgical inspection in a cohort of children and adults undergoing congenital aortic valvar surgery (18). The valvar morphology was categorized based on recent expert consensus of the normal and congenitally malformed aortic root (12). Each of the three aortic valvar commissures were marked in the short axis of the aortic root. Mid-diastolic measurements (70% R-R) were preferred when available, however, in the minority of cases involving only a peak systolic gated acquisition, measurements were performed in peak systole. The phase of the cardiac cycle where measurements were obtained was recorded. Angles were measured between each of the three sets of commissures with reference to the centroid of the trisinuate aortic root (X, right and non-coronary leaflet commissure; Y, right and left coronary leaflet commissure; Z, left and non-coronary leaflet commissure) (Figure 1A). In the short axis of the aortic virtual basal ring and sinus of Valsalva planes, a center bisecting plane cutting across the midline of one leaflet and continuing across the zone of apposition between the other two leaflets was obtained. The commissural height of this zone of apposition, which represents the height of the intervening interleaflet triangle, was qualitatively compared both to the plane of the sinutubular junction and to the other two commissural heights (Figures 1B–F) (12, 17). When available, the short axis cine of the aortic valve was visualized from the four-dimensional CTA cine to further assess the valvar morphology. The combined assessment of the commissural angles, comparison between the commissural heights, and visual dynamic assessment of the valve were used to distinguish the morphology of the aortic root and its valve (11, 12).

To assess for variability in the rotational position of the aortic root relative to the base of the left ventricle, a midline point was marked at the base of the inferoseptal recess roof (Point i), in line with the atrial septum, and compared to the angle with the non-coronary leaflet nadir. The aorto-ventricular angle was assessed from the coronal view as the angle between a horizontal line at the level of the basal left ventricle and the aortic valve annulus (21).

### Morphological method for diagnosing a bileaflet aortic valve

In those suspected to have leaflet fusion with leaflet and sinus asymmetry, the following findings supported the diagnosis of a congenital functionally bileaflet aortic valve:

the two suspected normal commissures deviating from their normal commissural angle of approximately 120–130 degrees,
and a commissural height of the suspected fused commissure significantly shorter than the two normal commissures, and significantly below the plane of the sinutubular junction (Figure 1) (12).

### Procedural details and outcomes

The type of transcatheter heart valve deployed was recorded. Requirement for PPI within 30-days following the procedure was recorded. Other common complications which were not consistently recorded and available in this retrospective chart review, such as paravalvar leak, were not assessed.

### Statistical analysis

Continuous variables were summarized as mean (standard deviation), and categorical variables as frequencies (percentages). Student's *t*-test and Pearson's Chi-squared test with continuity correction (where applicable) were performed to compare continuous and categorical variables across groups of interest, respectively. Linear models adjusted for CTA assessment phase and commissural angle width were regressed on anatomical variables to assess the difference between valve types. The receiver-operator characteristics (ROC) of the commissural angle were examined using a simple binary logistic model by regressing the angle of non-fused commissures on the judgement of the valve being bileaflet. To account for the potential differences in measurements made in systolic and diastolic phases, the assessed relationships were additionally adjusted for CTA assessment phase. A simple (unadjusted) binary logistic model was regressed to assess aortic leaflet morphology related to the occurrence of PPI, with adjusted and unadjusted models assessing the impact of prosthetic valve type.

An alpha level <0.05 was assessed to identify statistical relationships not due to chance. All statistical analyses were performed in R (22), and with the help of packages *pROC* (23) and *emmeans* package (24).

## Results

Four-hundred and thirty-three patients were included in the analysis with a mean (SD) age of 81.3 years (6.6), 53.8% female. Due to retrospective, multicenter design of the study, the quantitative CTA assessments were carried out in either diastole or systole as described in the methods: 270 cases were analyzed in diastole (62%), and another 163 (38%) were analyzed in systole. Utilizing our morphological method for diagnosis, 393 (90.8%) were determined to have trileaflet and 40 (9.2%) functionally bileaflet aortic valves with trisinuate aortic roots. No patient had a bileaflet valve with bisinuate aortic root. Based on the pre-procedural cardiac CT report, bileaflet aortic valves had been prospectively misdiagnosed as having a trileaflet valve in 32 of 40 cases (80%), while 4% of those with a trileaflet aortic valve were incorrectly classified as having a bileaflet valve. The baseline demographics, pre-procedural aortic virtual basal ring dimensions and prosthetic device type and size compared between these two groups are summarized in Table 1.

**Table 1:** Baseline demographics, pre-procedural rhythm electrocardiographic findings, aortic virtual basal ring dimensions and device selection.

Variable	Trileaflet aortic (*n* = 393)		Bileaflet aortic (*n* = 40)		*p*-value
	Mean/N	STD/%	Mean/N	STD/%	
Age (years)	81.5	6.6	79.5	6.0	0.0449
Sex (female)	215	55%	16	40%	0.1769
Atrial Fibrillation	81	21%	8	20%	1.0000
Diabetes Mellitus	101	26%	10	25%	1.0000
Chronic Kidney Disease	42	11%	6	15%	0.1671
Hypertension	273	69%	23	58%	0.2395
Right bundle branch block	36	9%	4	10%	1.0000
Left bundle branch block	25	6%	8	20%	0.0051
1st degree AV block	116	30%	19	48%	0.0282
Aortic VBR perimeter (mm)	75.1	7.3	81.2	9.9	0.0005
Aortic VBR major axis diameter (mm)	26.5	2.7	28.5	3.5	0.0010
Aortic VBR minor axis diameter (mm)	21.1	2.6	22.8	3.1	0.0015
Device (self-expanding valve)	240	61%	23	58%	0.7869
Device Type					0.1017
Acurate™	65	17%	2	5%	
Evolut™	93	24%	15	38%	
JenaValve™	5	1%	0	0%	
Myval™	17	4%	0	0%	
Navitor™	77	20%	6	15%	
Sapien™	136	35%	17	43%	
Device size (mm)	26.3	2.5	27.5	2.8	0.0181
CTA Assessment Phase					0.3809
Systolic	151	38%	12	30%	
Diastolic	242	62%	28	70%	

AV, atrioventricular block; CTA, computed tomography angiography; VBR, virtual basal ring.

Percentages may not add up to 100% due to rounding effect.

**Figure 2:**
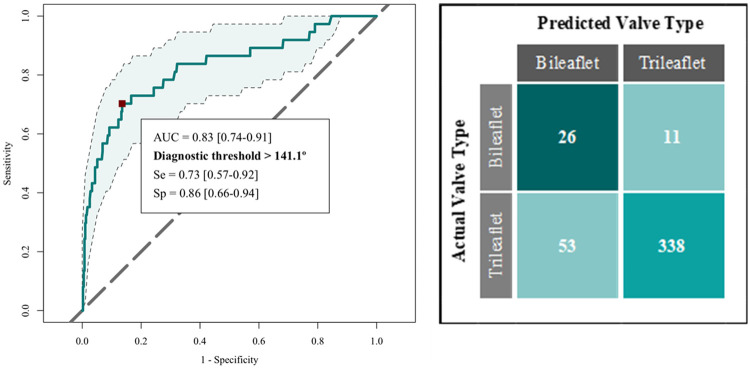
Left panel: receiver operator characteristics of the commissural angle in detecting the bileaflet aortic valve (AUC confidence intervals shaded in blue). Right panel: Confusion matrix for the same.

Thirty-four of the 40 patients (85%) with a bileaflet aortic valve had fusion between the right and left coronary leaflets, and the remaining 6 patients (17.5%) had fusion between the right and non-coronary leaflets. The mean (SD) commissural angle between the two normal commissures was 148.1 (18.3) degrees, with no significant difference related to fusion phenotype (*p* = 0.8656). This compared to a mean (SD) commissural angle in the trileaflet aortic valve (average of angles between X–Z and Z–Y commissures to correspond to the bileaflet valve phenotypes) of 126.0 degrees (13.3) (*p* *<* *0.0001*) (Table 2). The commissural angle demonstrated an area-under-the-curve of 0.83 [95% CI (0.74–0.91)] for identifying a functionally bileaflet aortic valve. A diagnostic angle threshold of 141.1 degrees had a sensitivity = 0.73 [95% CI (0.57–0.92)] and specificity = 0.86 [95% CI (0.66–0.94)] in identifying a bileaflet aortic valve (Figure 2). The angle between the non-coronary leaflet nadir and Point I and the aorto-ventricular angle (both adjusted for CTA assessment phase and commissural angle width) are reported in Table 3.

**Table 2:** Morphological characteristics of aortic valves.

Angle (degrees)	Trileaflet valves (*N* = 393)		Bileaflet valves (*N* = 40)				
	*µ*	sd	*µ*		sd		*p*-value
X-Y	110.4	11.7	99.0		13.1		<0.0001
Y-Z	123.7	11.8	116.6		18.5		0.0283
X–Z	126.0	13.3	144.4		21.3		<0.0001
Commissural^†^	*126*.*0*	*13*.*3*	*148*.*1*		*18*.*3*		<0.0001
Angle (degrees)	Trileaflet valves		Right/left coronary (*N* = 34)		Right/non-coronary (*N* = 6)		
			*µ*	sd	*µ*	sd	*p*-value
X–Y	Not Applicable		99.8	13.7	93.7	7.8	—
Y–Z			111.9	13.6	146.7	18.8	—
Z–X			148.3	18.6	119.6	22.4	—
Commissural^†^			148.3	18.6	146.7	18.8	0.8656

X, right and non-coronary leaflet commissure; Y, right and left coronary leaflet commissure; Z, left and non-coronary leaflet commissure.

† For trileaflet valves, the *commissural angle* was defined as the average of X–Z and Z–Y angles; for bileaflet valves with right/left coronary leaflet fusion the angle corresponds to X–Z, and for right/non-coronary leaflet fusion it corresponds to Y–Z.

X, right and non-coronary leaflet commissure; Y, right and left coronary leaflet commissure; Z, left and non-coronary leaflet commissure.

**Table 3:** Relationship of the aortic root to the left ventricle.

Variable	Trileaflet aortic valve		Bileaflet aortic valve		Adjusted difference	SE	*p*-value
	(*n* = 393)		(*n* = 40)				
	Mean	SD	Mean	SD			
Point I to non-coronary leaflet nadir angle (degrees)	−1.4	13.6	5.4	15.0	6.80	2.74	0.0064
Left ventricle to aortic angle (degrees)	49.2	10.3	52.4	12.0	3.19	1.60	0.1102

Means, standard deviations and difference of means (SE) are adjusted for CTA phase and commissural angle width; *p*-values are from the respective multiple linear models.

### Procedural outcomes

Fifteen of 40 patients (38%) with a bileaflet valve compared to 75 of 393 patients (19%) with a trileaflet valve had PPI requirement following TAVR (*p* = 0.0114) (Table 4). The unadjusted odds ratio for a bileaflet aortic valve requiring PPI was 2.54, 95% CI [1.25–5.01]). There was no relationship between balloon- vs. self-expanding device mechanism or size and the occurrence of PPI (Table 5). However, among specific devices, odds were lower for Acurate™ vs. Sapien™ [OR: 0.27, 95% CI (0.09–0.67), *p* = 0.0096], and comparable across remaining devices (Supplementary Table 1). This relationship remained significant after adjusting for the effect of the bileaflet valve [aOR: 0.29, 95% CI (0.10–0.73), *p* = 0.0157] (Supplementary Table 2).

**Table 4:** Comparative outcomes following transcatheter aortic valve replacement.

Outcome	Trileaflet aortic valve		Bileaflet aortic valve		Pearson's chi-squared *p*-value
	(*n* = 393)		(*n* = 40)		
	N	%	N	%	
Permanent pacemaker	75	19%	15	38%	0.0114
Left bundle branch block at discharge	75	19%	9	23%	0.3457
Left bundle branch block at 30-day post-op	37	9%	6	15%	0.1330

**Table 5:** Simple binary logistic regression on PPI occurrence in trileaflet and functionally bileaflet valves.

Predictor	Trileaflet aortic valve (*n* = 393)		Bileaflet aortic valve (*n* = 40)	
	OR	95% CI	OR	95% CI
Commissural angle (degrees)	1.00	[0.98–1.02]	1.02	[0.98–1.07]
Age (years)	1.00	[0.96–1.04]	1.10	[0.98–1.27]
Gender (Female)	0.62	[0.37–1.02]	1.29	[0.33–5.04]
Device type (self-expandible)	0.95	[0.57–1.59]	0.76	[0.21–2.81]
Device size (mm)	1.06	[0.96–1.17]	0.95	[0.72–1.21]
Major axis VBR diameter (mm)	1.08	[0.98–1.19]	0.92	[0.75–1.10]
Minor axis VBR diameter (mm)	1.05	[0.95–1.16]	0.87	[0.67–1.07]

PPI, permanent pacemaker implantation; VBR, virtual basal ring.

There were no significant differences in data missingness between trileaflet and bileaflet valves, with the exception of device size, leaflet angle measurements, and gender, all of which were missing slightly more often in cases with bileaflet valves (Supplementary Table 3).

## Discussion

This is the first systematic multi-center study to retrospectively utilize established cardiac CTA-based morphological assessment of the aortic root and its valve (11, 12) to accurately determine the true prevalence of congenital bileaflet (bicuspid) aortic valves in a population of patients with aortic stenosis who underwent TAVR. The applied approach has recently been validated in a surgical cohort and compared to intraoperative assessment (18), based on detailed CTA evaluation and morphological classification (11, 12, 18). In addition, this study provides a reliable understanding towards the relationship between a bileaflet valve with the post-TAVR requirement for PPI. The major findings are: 1) a poor diagnostic rate of detecting bileaflet aortic valves when utilizing standard CTA-based visual assessment; 2) an improved diagnostic rate of a bileaflet valve when quantitatively assessing the commissural angle; and 3) a significantly higher rate of PPI following TAVR in those with a bileaflet valve.

Those with a bileaflet aortic valve comprised almost one-tenth of patients undergoing TAVR. Of concern, over three-quarters of these patients were not identified by those reporting the cardiac CTA. Whether or not the performing interventionalist properly identified the valvar morphology prior to the procedure is not clear with the retrospective design of the current study. Over one-third of those with a bileaflet aortic valve required PPI following TAVR, which was over double the incidence of the larger cohort with trileaflet valves (39% vs. 19%, respectively). The current study was not able to properly assess for other common post-procedural complications, such as paravalvar leak, due to its retrospective design. This study, however, emphasizes the need for the improved delineation of aortic valvar morphology preceding TAVR in order to properly determine related procedural risks and complications. This understanding will in turn guide procedural and technological improvements specific to patients with these not uncommon valvar phenotypes referred for TAVR.

While comparison could be made to other studies which have investigated this same outcome in bileaflet valves, this multi-center study raises concern towards the current real-world detection rate in prospectively identifying these relatively common congenitally malformed aortic valves prior to any TAVR procedure, questioning the accuracy in prior reports. A similar experience in the common misdiagnosis of adults with congenitally malformed aortic valves prior to referral into an international congenital aortic valvar surgical referral center has been reported with validation on direct intraoperative inspection (11, 18). In those undergoing TAVR, detection may further be hindered in the setting of concomitant calcific aortic valvar disease, where calcifications may actually cause acquired fusion between leaflets. Quantitative assessment of the angle between the two normal commissures may improve the detection rate of congenital functionally bileaflet aortic valves. Specifically, a diagnostic angle greater than approximately 140 degrees has a sensitivity of 0.73 and specificity of 0.86. This diagnostic method may further be improved by assessing the commissural height of the suspected fused commissure with comparison to that of the normal commissures (10–12, 17, 25), in combination to visual inspection of the suspected fused zone of apposition in systole (see Figure 1). Depending solely on the latter, which presumably was the primary method used by those prospectively assessing the cardiac CTA prior to the TAVR procedure, is limited by the issue of through-plane motion of a three-dimensional structure through a two-dimensional plane of imaging and the difficulties mentioned in the valve with significant calcific disease (11, 12, 18).

In those with a bileaflet valve, there were significant differences in how the aortic root is positioned relative to the base of the left ventricle. This was reflected in a measurement of its rotational position (Point I to non-coronary leaflet nadir angle) and the angle of the long axis of the left ventricle relative to the aortic root. These findings support that the congenital aberration underyling the bileaflet aortic valve not only impacts the leaflets, but also how the aortic root itself is positioned relative the left ventricular outflow tract. These anatomical differences may contribute towards the increased post-procedural risks seen following TAVR, including both the increased risks for conduction damage and paravalvar leak (5).

### Limitations

The current study is limited by the smaller subset of patients with bileaflet valves. In addition, a fair number of patients in both trileaflet and bileaflet cohorts had missing data (reported in Supplementary Table 1) with no significant differences between trileaflet and bileaflet cohorts, with the exception of device size and the angle between the left ventricle and aortic root. This same limitation prohibited the ability to assess for other post-procedural risk factors which were not consistently documented. Furthermore, future studies incorporating assessment of commissural angles should aim to determine the reproducibility of these proposed methods aiding in morphological assessment of the aortic valve.

## Conclusion

Bileaflet aortic valves are common in older adults undergoing TAVR, though often misdiagnosed by traditional methods of dynamic short axis visualization. The diagnostic accuracy may be improved with assessment of the commissural angle and comparison of commissural heights. In those with a bileaflet valve, the rate of PPI following TAVR is almost doubled when compared to trileaflet aortic valves. Significant differences are seen in the position of aortic root relative to the left ventricle between these trileaflet and bileaflet aortic leaflet morphologies. These anatomical differences may contribute towards the increased risks for conduction damage and other complications following TAVR in those with a bileaflet aortic valve. Future studies using this morphological diagnostic method as a starting point in the proper assessment of procedural risk factors in this higher risk TAVR population are required to further validate this approach.

## Data Availability

The raw data supporting the conclusions of this article will be made available by the authors, without undue reservation.
