# Supplementary material for: Methods for improved bileaflet aortic valve detection prior to transcatheter aortic valve replacement
Source: Front Cardiovasc Med. 2026 Feb 23;13:1755835. doi: 10.3389/fcvm.2026.1755835 (PMC12967922; doi:10.3389/fcvm.2026.1755835)
Supplement: Supplementary file 1 [file Datasheet1.docx]

**Supplemental Table 1.** Device selection related to occurrence of permanent pacemaker insertion following transcatheter aortic valve replacement.

| **Device** | **All Patients** | | | | | | **Trileaflet** | | | | | | **Bileaflet** | | | | | |
| --- | --- | --- | --- | --- | --- | --- | --- | --- | --- | --- | --- | --- | --- | --- | --- | --- | --- | --- |
|  | **(N = 433)** | | | | | | **(N = 393)** | | | | | | **(N = 40)** | | | | | |
|  | **Without Permanent Pacemaker (n=343)** | | | **With Permanent Pacemaker (n=90)** | | | **Without Permanent Pacemaker (n=318)** | | | **With Permanent Pacemaker (n=75)** | | | **Without Permanent Pacemaker (n=25)** | | | **With Permanent Pacemaker (n=15)** | | |
|  | **N** | | **%** | **N** | | **%** | **N** | | **%** | **N** | | **%** | **N** | | **%** | **N** | | **%** |
| *Sapien™* | 118 | /153 | *77%* | 35 | /153 | *23%* | 108 | /136 | *79%* | 28 | /136 | *21%* | 10 | /17 | *59%* | 7 | /17 | *41%* |
| *Evolut™* | 87 | /108 | *81%* | 21 | /108 | *19%* | 77 | /93 | *83%* | 16 | /93 | *17%* | 10 | /15 | *67%* | 5 | /15 | *33%* |
| *Navitor™* | 58 | /83 | *70%* | 25 | /83 | *30%* | 55 | /77 | *71%* | 22 | /77 | *29%* | 3 | /6 | *50%* | 3 | /6 | *50%* |
| *Accurate™* | 62 | /67 | *93%* | 5 | /67 | *7%* | 60 | /65 | *92%* | 5 | /65 | *8%* | 2 | /2 | *100%* | 0 | /2 | *0%* |
| *Myval™* | 15 | /17 | *88%* | 2 | /17 | *12%* | 15 | /17 | *88%* | 2 | /17 | *12%* | 0 | /0 | *–* | 0 | /0 | *–* |
| *JenaValve™* | 3 | /5 | *60%* | 2 | /5 | *40%* | 3 | /5 | *60%* | 2 | /5 | *40%* | 0 | /0 | *–* | 0 | /0 | *–* |

**Supplemental Table 2.** Association of specific devices and the occurrence of PPI, adjusted for the effect of the native valve morphology (trileaflet vs. bileaflet).

| **Predictor** | **aOR** | **95% CI** | | ***p-value*** | |
| --- | --- | --- | --- | --- | --- |
| ***Reference level**** | 1.00 | – | | – | |
| ***Device*** |  |  |  |  | |
| *Evolut™* | 0.79 | [0.42– | 1.44] | 0.4445 | |
| *Navitor™* | 1.52 | [0.82– | 2.79] | 0.1757 | |
| *Accurate™* | 0.29 | [0.10– | 0.73] | **0.0157** | |
| *Myval™* | 0.51 | [0.08– | 1.92] | 0.3829 | |
| *JenaValve™* | 2.53 | [0.32– | 15.9] | 0.3202 | |
| ***Bileaflet aortic valve*** | 2.45 | [1.19– | 4.92] | **0.0128** | |
| *Interpret reference level as trileaflet aortic valve implanted with Sapient™ | | | | |  |

**Supplemental Table 3.** Missingness in Data

|  | **Trileaflet** | | **Bileaflet** | | **Chi-Squared p-value** |
| --- | --- | --- | --- | --- | --- |
|  | **(n = 393)** | | **(n = 40)** | |  |
| **Variable** | **n** | **%** | **n** | **%** |  |
| *Age (years)* | 0 | 0% | 0 | 0% | – |
| *Sex (female)* | 2 | 1% | 2 | 5% | 0.0499 |
| *Atrial Fibrillation* | 77 | 20% | 10 | 25% | 0.5445 |
| *Diabetes Mellitus* | 78 | 20% | 10 | 25% | 0.5719 |
| *Chronic Kidney Disease* | 222 | 56% | 27 | 68% | 0.2403 |
| *Hypertension* | 77 | 20% | 10 | 25% | 0.5445 |
| *Right bundle branch block* | 8 | 2% | 1 | 3% | 1.0000 |
| *Left bundle branch block* | 8 | 2% | 1 | 3% | 1.0000 |
| *1^st^ degree AV block* | 8 | 2% | 1 | 3% | 1.0000 |
| *Aortic VBR perimeter* | 10 | 3% | 0 | 0% | 0.6396 |
| *Aortic VBR major axis diameter* | 12 | 3% | 0 | 0% | 0.5384 |
| *Aortic VBR minor axis diameter* | 12 | 3% | 0 | 0.00 | 0.5384 |
| *Device (self-expanding valve)* | 0 | 0% | 0 | 0% | – |
| *Device Type* | 0 | 0% | 0 | 0% | – |
| *Device size* | 5 | 1% | 4 | 10% | 0.0019 |
| *CTA Assessment Phase* | 0 | 0% | 0 | 0% | – |
| *X-Y* | 2 | 1% | 3 | 8% | 0.0015 |
| *Y-Z* | 3 | 1% | 3 | 8% | 0.0057 |
| *X-Z* | 2 | 1% | 3 | 8% | 0.0015 |
| *Point I to non-coronary leaflet nadir angle* | 3 | 1% | 1 | 3% | 0.8209 |
| *Left ventricle to aortic angle* | 57 | 15% | 11 | 28% | 0.0543 |
| *Permanent Pacemaker* | 0 | 0% | 0 | 0% | – |
| *Left Bundle Branch Block at Discharge* | 83 | 21% | 14 | 35% | 0.0708 |
| *Left Bundle Branch Block at 30-day post-op* | 153 | 39% | 21 | 53% | 0.1341 |

AV, atrioventricular; CTA, computed tomography angiography; VBR, virtual basal ring
